# Book Review

**DOI:** 10.2188/jea.17.140

**Published:** 2007-07-18

"Those who learn from experience are the foolish, whereas those from history are the wise." This is said to be an epigram by Otto von Bismarck, called as the Iron Chancellor. Because many epidemiologists try to be wise, many of them start the class of epidemiology with "Snow on Cholera," including me. (Unfortunately, I do not know the original German of the epigram.)

Fortunately, Professor Kunio Aoki, who is a previous president of the International Epidemiological Association (IEA), reviewed the history of epidemiology in Japan till the end of World War II.^1^ He is now trying to review the history after the War as well, and it will appear on the Journal soon.

Professor Walter W. Holland, who is also a previous president of the IEA, had been editing the history of the modern epidemiology in global with Professor Jørn Olsen, the present president of IEA, and Professor Charles du V. Florey. The fruit has been published as "The Development of Modern Epidemiology: Personal Reports from Those Who Were There" by the Oxford University Press in this spring. Professor Holland introduced a part of this book in his special lecture on the 17th annual meeting of the Japan Epidemiological Association in Hiroshima on January 2007. At that time, I saw only the galley proof from the Professor, and was impressed because of its profound contents. This is not "Personal Reports!", but a book compiled all the history in recent 50 years. Now I get the book, and my impression about the book becomes greater and greater.

This book has 5 sections; (l)History and setting the scene, (2)Specific disease areas of concern, (3)Applications and role of epidemiology in related domains, (4)Methodology, and (5)Regions and countries. Each section consists of 4 to 14 parts, and every part is written by great epidemiologists. For example, the part of "Development of the epidemiology of cancer," the first part of the Section 2 (Specific disease areas of concern), is by Sir Richard Doll. As we know, Sir Richard passed away in July 2005, and this is his last work. The 8th part of the Section 5 (Regions and countries), "Epidemiological methods: a view from north Asia/Japan," is written by Professor Aoki and Dr. Itsuzo Shigematsu, a honorary member of the IEA. Every part has the preferable number of references concerning the part so that readers wishing to know more about the issue can learn in details. Personally, "Theoretical developments" part on the Section 4 (Methodology) written by Professor Olli S. Miettinen is the most interesting because I learned statistics in epidemiology with tears through his book "Theoretical Epidemiology" published in 1985. If a young epidemiologist wishes to learn theories of the epidemiologic methods, he/she must read this part and all of the references here.

The most interesting segments of the book exist on the top of the parts. Not all, but many parts have "Personal experience" segments, and here many authors present his/her personal involvement including curriculum vitae. Everyone is different, and so are epidemiologists! I can get information about background of the leading epidemiologists whom I meet on the Councillor Meeting of the IEA. I cannot ask it directly, and this information is helpful for me.

If an epidemiologist would like to be a wise one, in particular for young epidemiologists, this book is essential. If you read one of the parts described by great epidemiologists, your work must be changed.

Finally, I would quote the last part of the Part 1 "Introduction" written by the 3 editors, and close this book review. "This book certainly illustrates the enormous contributions epidemiology has made in the past 50 or more years, both in methods and as well as applications to health improvements. The IEA can be proud to have fostered this activity and provides an excellent foundation for the future advancement of the discipline."
